# Legal solutions for medical profession

**Published:** 2008

**Authors:** Sarvesh Chandra

**Affiliations:** St. Mathews Scientific Research Foundation, C/o Muktesh Hospital, A - 12, Ekta Nagar, Stadium Road, Bareilly, Uttar Pradesh - 243122, India. E-mail: smsrf@rediffmail.com, Web: www.mylegaldoctor.com

A comprehensive book, probably first time in India, on a wide variety of legal issues concerning medical profession which is meant to provide necessary legal knowledge for solving day-to-day problems of doctors, nursing homes, hospitals, blood banks, and other medical establishments.

It is an aid to the medical profession for protection against exploitation and harassment. The topics covered in this book include Medical Council of India, dental/homeopathy/Indian medicines/nursing/pharmacy/rehabilitation councils. MTP, PCPNDT, transplantation of human organs, mental health, blood banks, biomedical waste, registration of births and deaths, epidemic diseases, consumer protection, human rights, right of information, electricity, development authority, municipal tax, minimum wages and related labor laws, employee's provident fund and related schemes, criminal procedure code, income tax search, and surveys with PAN/TAN, professional indemnity insurance, health insurance, Supreme Court judgments, forms for registering complaints against harassment with Lok-Ayukta and National Human Rights Commission, Fundamental Rights, citizenship, Red Cross, etc.

This book also contains copies of official documents which are useful to settle day-to-day problems. However, these documents mainly relate to state of Uttar Pradesh, but their application can be extrapolated to other states as well as most of the laws are similar if not identical in almost all the states.

The writer is himself a doctor and hence is well aware about the ignorance of doctors in general about their responsibilities and rights while being subjected to laws of the land. In this context, the book is a legal companion for all the people connected with medical profession. It will serve as a reference book whenever faced with any legal or administrative problems.

